# Anti-Inflammatory Effect of Geniposide on Osteoarthritis by Suppressing the Activation of p38 MAPK Signaling Pathway

**DOI:** 10.1155/2018/8384576

**Published:** 2018-02-26

**Authors:** Yuan Chen, Kangquan Shou, Chunlong Gong, Huarui Yang, Yi Yang, Tongzhu Bao

**Affiliations:** ^1^Department of Emergency and ICU, The First Clinical Medical College, China Three Gorges University, Yichang, Hubei 443002, China; ^2^Department of Orthopaedics, The First Clinical Medical College, China Three Gorges University, Yichang, Hubei 443002, China

## Abstract

It has been suggested that the activation of the p38 mitogen activated protein kinases (MAPKs) signaling pathway plays a significant role in the progression of OA by leading to the overexpression of proinflammatory cytokines, chemokines, and signaling enzymes in human osteoarthritis chondrocytes. However, most p38 MAPK inhibitors applied for OA have been thought to be limited due to their potential long-term toxicities. Geniposide (GE), an iridoid glycoside purified from the fruit of the herb, has been widely used in traditional medicine for the treatment of a variety of chronic inflammatory diseases. In this study, we evaluated the inhibition effect of geniposide on the inflammatory progression of the surgically induced osteoarthritis and whether the protective effect of geniposide on OA is related to the inhibition of the p38 MAPK signaling pathway.* In vitro*, geniposide attenuated the expression of inflammatory cytokines including interleukin-1 (IL-1), tumor necrosis factor (TNF-*α*), and nitric oxide (NO) production as well as matrix metalloproteinase- (MMP-) 13 in chondrocytes isolated from surgically induced rabbit osteoarthritis model. Additionally, geniposide markedly suppressed the expression of IL-1, TNF-*α*, NO, and MMP-13 in the synovial fluid from the rabbits with osteoarthritis. More importantly, our results clearly demonstrated that the inhibitory effect of geniposide on surgery-induced expression of inflammatory mediators in osteoarthritis was closely associated with the suppression of the p38 MAPK signaling pathways. Our study demonstrates that geniposide may have therapeutic potential to serve as an alternative agent for the p38 MAPK inhibition for the treatment of OA due to its inherent features of biological activities and low toxicity as a traditional Chinese medicine.

## 1. Introduction

Osteoarthritis (OA) is a degenerative joint disease that leads to painful inflammation of the joints and surrounding tissues, with an ever increasing incidence rate every year in elderly populations [1, 2]. It is widely accepted that a variety of factors, including aging, genetic factor, trauma, obesity, gender, and hormone, may contribute to development of OA [3]. More importantly, the unbalanced status of proinflammatory and anti-inflammatory signaling pathways has been considered as a major cause of OA [4, 5]. The overexpressed proinflammatory cytokines which inhibited the cartilage matrix synthesis and chondrocytes proliferation such as interleukin-1 (IL-1) and tumor necrosis factor-alpha (TNF-*α*) are thought to participate in the pathophysiology progression of OA [6–8]. Therefore, a variety of anti-inflammatory drugs such as nonsteroidal anti-inflammatory drugs (NSAIDs) have been used to attenuate the overinflammatory status related to the articular cartilage injury at early stage of OA. However, most of these drugs suffered from obvious drawbacks including the gastrointestinal haemorrhage [1] and renal impairment [9]. Additionally, so far none of these drugs can address the problem to slow the progression of the autoimmune disease and cure the OA. Hence, an alternative and effective therapeutic drug urgently needs to be developed to solve this issue.

*Gardenia jasminoides* Ellis (Rubiaceae), known as “zhizi,” a traditional medicinal herb, has been widely used for rheumatism, thrombotic, hepatic disorders, and neurodegenerative disorders due to its naturally anti-inflammatory and antioxidative properties [10, 11]. Geniposide (GE), an iridoid glycoside purified from the fruit of the herb, has been proved as the major active ingredient of* Gardenia jasminoides*. Previous studies showed that GE possessed antiangiogenic [12], anti-inflammatory [13, 14], antivascular injury [15], and protective effects against hepatic damage [16]. In our previous* in vitro* study, GE was demonstrated to be able to suppress the apoptosis of rat chondrocytes by reducing the concentration of inflammatory mediators such as NO in the culture supernatants, leading to the enhanced cell proliferation and recovery of chondrocytes [17]. Nevertheless, the specific protective mechanism of geniposide on the degeneration of articular cartilage in the OA process still remains unclear.

Mitogen activated protein kinase (MAPK), one of intracellular serine-threonine protein kinase superfamily members, is the central node of multiple signal transduction pathways [18]. Normally, the MAPKs pathway consists of three signal cascades' pathway: c-Jun N-terminal kinase (JNK), p38, and extracellular signal-regulated kinase (ERK) [19]. A large number of experiments suggested that the p38 signaling pathway plays a key role in the progress of considerable human diseases, especially for the development of OA [20–23]. Activation of the p38-MAPK signaling pathway may lead to the expression of proinflammatory cytokines, chemokines, MMPs, and signaling enzymes (COX-2) in human osteoarthritis chondrocytes [24]. Blockage of p38 MAPK with p38 inhibitor could suppress the chondrocytes apoptosis and decrease the downstream inflammatory cytokine production, preventing the recruitment of other inflammatory cells that may contribute to degradation of bone and cartilage [25]. Previous study performed in PC12h cells (rats pheochromocytoma cells) has proposed that geniposide may have certain impact on the neurite outgrowth by induction of the MAPK phosphorylation with the help of nerve growth factor (NGF) [26]. Thus, in this study, we hypothesize that geniposide is able to inhibit the expression and production of inflammatory mediators in OA chondrocytes and OA models, and this inhibitory effect may be closely related to the alleviation of activation of the p38 MAPK signaling pathway.

## 2. Materials and Methods

### 2.1. Materials

SB203580 (4-(4-fluorophenyl)-2-(4-methylsulfinylphenyl)-5-(4-pyridyl) 1H-imidazole) was purchased from Biomol (Plymouth, PA, USA). The free GE solution was prepared by suspending pure GE powder (provided by Yuanye Biotechnology, Shanghai, China) in 0.5% sodium carboxymethyl cellulose (CMC-Na).

### 2.2. Induction of Osteoarthritis (OA) in Rabbit Model

Thirty New Zealand white rabbits (3-month-old, 2.0 ± 0.2 Kg, Certificate SCXK 2011-0011) were purchased from the Wuhan WanQianJiaXing Biotechnology Co. Ltd., Wuhan, China. Animals were housed under a 12 h light/dark cycle (lights on from 08:00~20:00) at constant temperature (23°C) and humidity (55%). All the experimental procedures were performed and approved by the Institutional Animal Care and Use Committee (IACUC) of Three Gorges University. OA model was surgically induced by the modified “Hulth method” [27]. All the surgical procedures were performed under sterile conditions. Anesthesia was performed prior to the surgery by injecting with 1.0 ml/kg of pentobarbital solution (3% pentobarbital solution with physiological saline, 1 : 1) into the ear vein. An incision with 5 cm length was made on the anteromedial skin. Then, the medial meniscus, the medial collateral ligament, and the anterior cruciate ligament (ACLT) were resected with the patella preserved. The surgery was considered successful when positive sign was observed by the anterior drawer test and the medial lateral stress test. Next, the joint was washed successively with diluted povidone iodine solution, hydrogen peroxide solution, and sterile saline solution. Then the capsule, the synovium, and the skin were then closed with 4.0 interrupted vicryl. All rabbits were injected daily with penicillin intramuscularly with a dose of 400,000 units each for one week. All procedures were performed by a single surgeon in a blinded fashion. All rabbits were kept in single-subject cages.

### 2.3. Isolation, Culture, and Characterization of Articular Chondrocytes from OA Rabbit

In order to harvest OA rabbit articular chondrocytes, at the end of two months after the operation, six rabbits were sacrificed with an overdose of hydrochloride and xylazine, and then the cartilage tissues were collected from knee joints and sliced into pieces approximately 1 mm^3^ and transferred into centrifuge tube with the DMEM and then centrifuged at 800 rpm for 10 min. Then the cartilage pieces were transferred into culture dish and 0.2% collagenase II (Sigma, USA) was added for the cartilage matrix dissociation at 37°C incubator. After 45 min, FBS-DMEM medium, containing 10% (v/v) heat-inactive fetal bovine calf serum, 100 U/ml penicillin, and 100 *μ*g/ml streptomycin, was added to the incubator to stop the digestion. Then the tissues were transferred into centrifuge tube and centrifuged at 1100 rpm for 5 min. The supernatant was aspirated and the chondrocytes were resuspended in addition to 10 ml FBS-DMEM medium. The chondrocytes harvested from healthy rabbits were considered as normal.

### 2.4. Treatment of OA Models

At the end of two months, twenty-four rabbits with OA were divided randomly into four groups. For geniposide group, an aqueous solution of geniposide (40 mg/kg) was delivered daily by intragastric gavage for two weeks. For SB203580 (a reliable and classic p38 MAPK inhibitor [28]) group, an aqueous solution of SB203580 (10 mg/kg) was delivered daily by intragastric gavage for two weeks. For geniposide + SB203580 group, both the geniposide and the SB203580 with the same dose as geniposide and SB203580 group were administrated by intragastric gavage for two weeks. For control groups, an equivalent amount of aqueous solution was delivered by intragastric gavage for two weeks. All animals were housed individually.

On day 14 after the drug administration, the synovial fluid of the knee joint was collected and the cartilage tissues were harvested. For histological analysis, harvested specimens were immediately snap frozen in Optimal Cutting Temperature compound (OCT compound) (Sakura Finetek, USA) or fixed in 4% paraformaldehyde overnight and embedded in paraffin for haematoxylin and eosin (H&E) staining, immunohistochemistry, and immunofluorescence analyses. All analyses were evaluated by two pathologists in a blinded fashion.

### 2.5. MTT Assay

To evaluate the safety and the biocompatibility of geniposide, MTT assay was performed. Geniposide powder was dissolved in DMSO and then diluted with 10% FBS-DMEM to the final concentration of 10 *μ*g/ml, 20 *μ*g/ml, 40 *μ*g/ml, 80 *μ*g/ml, and 160 *μ*g/ml, respectively. The OA chondrocytes at the logarithmic growth phases were collected and seeded in 96-well plates with a density of 5 × 10^3^ cells per well. After 24 h, the medium of 96-well plates was replaced with 100 *μ*L of medium containing different concentration of GE and then incubated for 24 h, 48 h, and 72 h, respectively. Next, MTT (10 *μ*L, 0.5 mg/mL) solution was added to each well and then incubated for 4 h at 37°C. The supernatant was removed and the residues were lysed with 200 *μ*L of dimethyl sulfoxide (DMSO). The absorbance value was recorded at 490 nm using a microplate reader while the untreated cells were used as a control.

### 2.6. Toluidine Blue Staining

The toluidine blue staining was performed to indicate the presence of glycosaminoglycans and identify the discrepancy existing between the normal chondrocytes and OA chondrocytes [29]. Approximately 4 × 10^5^ cells' chondrocytes (Passage 2) were cultured into a sterile cover glass within a 6-well plate. When chondrocytes became adherent, the cover glass was taken out and washed with 5% PBS for three times. Then the cells were treated with 70% ethanol for 20 min and then washed extensively with 5% PBS (5 min ×3). Then the chondrocytes were stained with 0.04% toluidine blue for 30 min and then washed by 100% ethanol and cleared by xylene, finally mounted, and observed under microscope.

### 2.7. Immunohistochemistry (IHC) Staining

Immunohistochemistry was performed to assess the deposition of collagen II in the chondrocytes, according to manufacturer's instructions. Briefly, the slides of cells or tissue sections described above were incubated with primary antibodies (anti-collagen type II, 1 : 150, and anti-CD31, 1 : 100, Abcam, United Kingdom) overnight at 4°C and then incubated with HRP-coupled secondary antibodies (Aspen, China). Staining was performed using diaminobenzidine (brown).

### 2.8. Enzyme-Linked Immunosorbent Assay (ELISA)

To evaluate the inflammatory status of the knee joint, the levels of IL-1*β* and TNF-*α* in serum were measured by ELISA kits according to the manufacturer's instructions. Absorbance was measured using a microplate reader at 450 nm (Sigma, St. Louis, MO, USA).

### 2.9. Determination of NO Content

The determination of total NO content in the supernate of the cultured OA chondrocytes 24 h after incubation with geniposide solution (80 *μ*g/ml) or harvested specimens was measured according to the Griess reaction [30]. The concentration of the reaction product and the NO concentration maintained a linear relationship, and the maximum absorption peak was detected at a wavelength of 550 nm.

### 2.10. Western Blot Analysis

Total protein was isolated from chondrocytes or harvested specimens with a Total Protein Extraction Kit (Aspen, China). Equal amounts of protein from cell or tissue lysates were loaded onto a 5% SDS polyacrylamide gel (Aspen, China) and transferred to a polyvinylidene fluoride (PVDF) membrane (Millipore, USA). Membranes were blocked with 5% BSA in TBS and then incubated with primary antibodies against IL-1*β* (1 : 500), TNF-*α* (1 : 1000), MMP-13 (1 : 500), p38 (1 : 2000), p-p38 (1 : 1000), and *β*-actin (1 : 10000) overnight at 4°C. Next, an HRP-conjugated secondary antibody was applied (1 : 10000) and detected with the Immobilon Western Chemiluminescent HRP Substrate system (Millipore, USA). All of the antibodies were purchased from Abcam Inc. (United Kingdom).

### 2.11. Real-Time Quantitative PCR

After incubation with geniposide solution (80 *μ*g/ml) for 24 h, the supernate of the cultured OA chondrocytes or the harvested specimens from the animal models were collected. Then, total RNA was extracted via the TaKaRa MiniBEST Universal RNA Extraction Kit (Takara Bio, Japan), following the manufacturer's instructions. cDNA was synthesized using the PrimeScript™ II First Strand cDNA Synthesis Kit (Takara Bio, Japan), according to the manufacturer's protocol. PCR conditions comprised an initial step of denaturation for 1 min at 95°C, followed by a total of 40 cycles of 15 s at 95°C, 20 s at 58°C, and 20 s at 72°C. After normalization against the housekeeping gene *α*-tubulin, the expression of genes of interest (Table 1) was measured using the 2^−ΔΔCt^ method.

### 2.12. Statistical Analysis

All values are expressed as mean ± SD. Statistical significance was measured using Student's unpaired *t*-test (two-tailed). The difference among groups was detected using the one-way analysis of variance (ANOVA) test. A *p* value < 0.05 was considered statistically significant.

## 3. Results

### 3.1. Characterization of OA Chondrocytes

**Figure 1:**
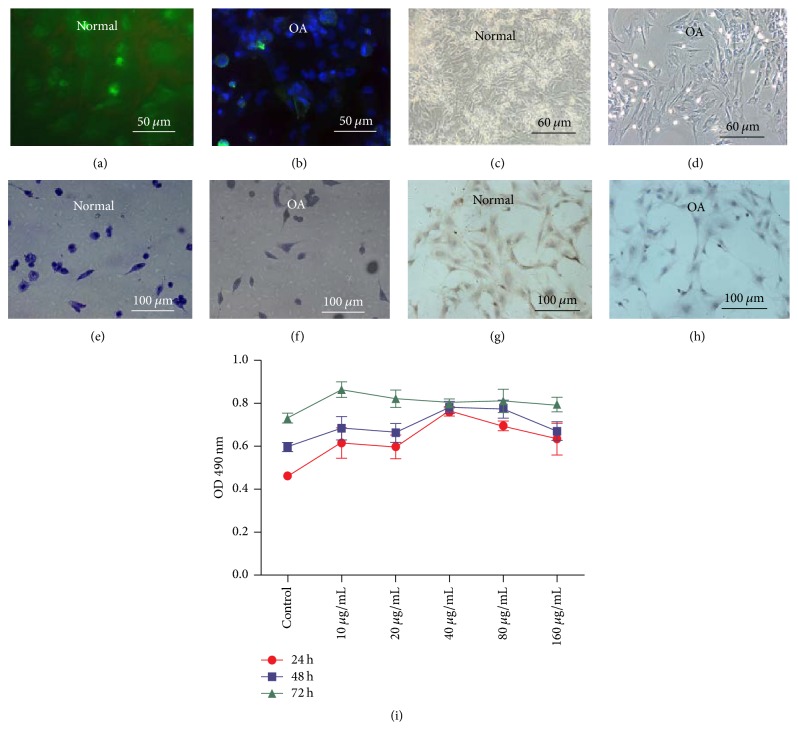
*Characterization of normal and OA chondrocytes and the biocompatibility of geniposide*. The positive expression of collagen II was demonstrated by immunofluorescence staining (a) compared with OA chondrocytes (b). The light microscopy revealed morphology of the normal and OA chondrocytes cultured in plates (c, d), identifying that the cells harvested from rabbits were chondrocytes. Notably, OA chondrocytes (f) were shown with slight blue color compared with normal chondrocytes (e) by using toluidine blue staining. The immunohistochemistry staining of collagen II showed that the positive staining with brown was observed at cytoplasm of normal chondrocytes (g), while the expression of collagen II was decreased in OA chondrocytes (h). (i) The MTT assay showed that geniposide with 10–160 *μ*g/mL did not exhibit a cytotoxic effect on OA chondrocytes.

The positive expression of collagen II was demonstrated in the normal chondrocytes by immunofluorescence staining (Figure 1(a)) compared with OA chondrocytes (Figure 1(b)). The light microscopy revealed morphology of the normal and OA chondrocytes cultured in plates (Figures 1(c) and 1(d)), identifying that the cells harvested from rabbits were chondrocytes. As shown in Figure 1(f), the cytoplasm and nucleus of OA chondrocytes were shown with slight blue color compared with normal chondrocytes (Figure 1(e)) by using toluidine blue staining. Additionally, the immunohistochemistry (IC) staining of collagen II showed that the positive staining with brown was observed at cytoplasm of normal chondrocytes (Figure 1(g)), while the expression of collagen II was decreased in OA chondrocytes (Figure 1(h)).

### 3.2. Effects of Geniposide on OA Chondrocytes Viability

Our study showed that geniposide at 10–160 *μ*g/mL did not show a cytotoxic effect on OA chondrocytes at 24, 48, and 72 h after treatment with the indicated concentrations of geniposide (Figure 1(i)), demonstrating the feasibility of clinical application of geniposide.

### 3.3. Effects of Geniposide on IL-1*β*, TNF-*α*, and MMP-13 Expression and Production in OA Chondrocytes

**Figure 2:**
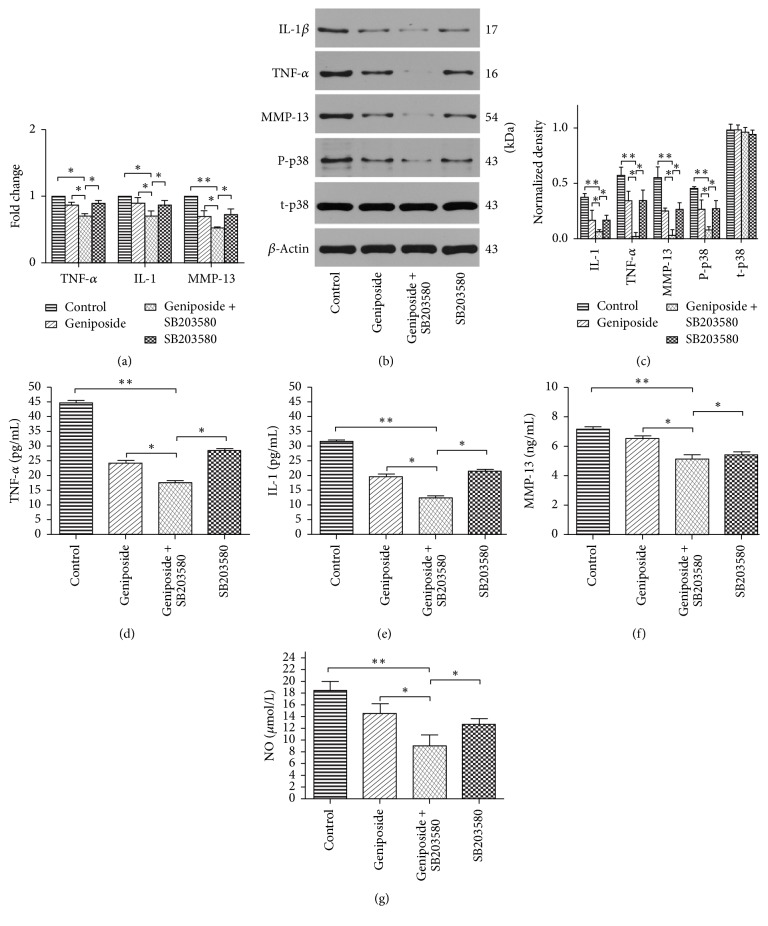
*Effects of geniposide on inflammatory mediators and p38 MAPKs signaling pathway in OA chondrocytes in vitro*. (a) qRT-PCR analysis of IL-1*β*, TNF-*α*, and MMP-13 gene expression in OA chondrocytes incubated with geniposide and SB203580 as well as the combination of these two drugs, respectively. The data of control were considered as 1. (b) Western blotting of IL-1*β*, TNF-*α*, MMP-13, p-p38 (phosphorylation of p38), and t-p38 (total p38) protein expression among four groups. (c) Quantification of western blotting. The expression level of TNF-*α*, IL-1*β*, MMP-13, and NO in the supernatants of the OA chondrocytes (d–g) treated with geniposide and SB203580 as well as the combination of these two drugs, respectively. Data is given as the mean ± SD; ^*∗*^*p* < 0.05 and ^*∗∗*^*p* < 0.01.

**Figure 3:**
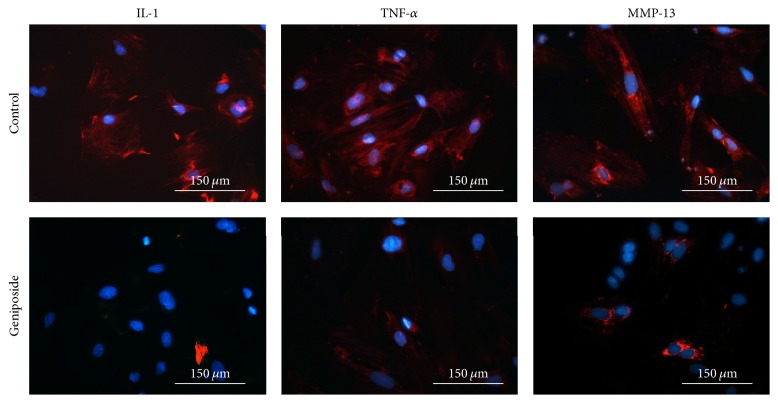
*The anti-inflammatory effect of the geniposide*. The immunofluorescent staining demonstrated that OA chondrocytes incubated with geniposide exhibited decreased expression of IL-1*β*, TNF-*α*, and MMP-13 compared with control group.

After 24 h of incubation with geniposide and SB203580, the western blotting results exhibited markedly decreased protein expression compared with the control group for IL-1*β* (0.0620 ± 0.0190, *p* < 0.01), TNF-*α* (0.0210 ± 0.037, *p* < 0.01), and MMP-13 (0.032 ± 0.0481, *p* < 0.01) (Figures 2(b) and 2(c)); meanwhile the inhibition of geniposide alone was also demonstrated as shown in Figures 2(b) and 2(c). Similarly, real-time qPCR analysis (Figure 2(a)), immunofluorescence staining (Figure 3), and ELISA (Figures 2(d)–2(f)) confirmed the same results as those observed in western blotting analyses.

### 3.4. Effect of Geniposide on p38 MAPKs Pathway in OA Chondrocytes

Our findings exhibited that geniposide was able to attenuate the phosphorylation of p38 and the inhibition effect could be synergized by the p38 MAPKs inhibitor SB203580 (Figures 2(b) and 2(c)), suggesting that favorable effects of geniposide on OA are achieved by a stronger inhibitory effect on p38 MAPKs signaling pathway.

### 3.5. Effects of Geniposide on IL-1*β*, TNF-*α*, and MMP-13 Expression and Production in the OA Knee Joint

**Figure 4:**
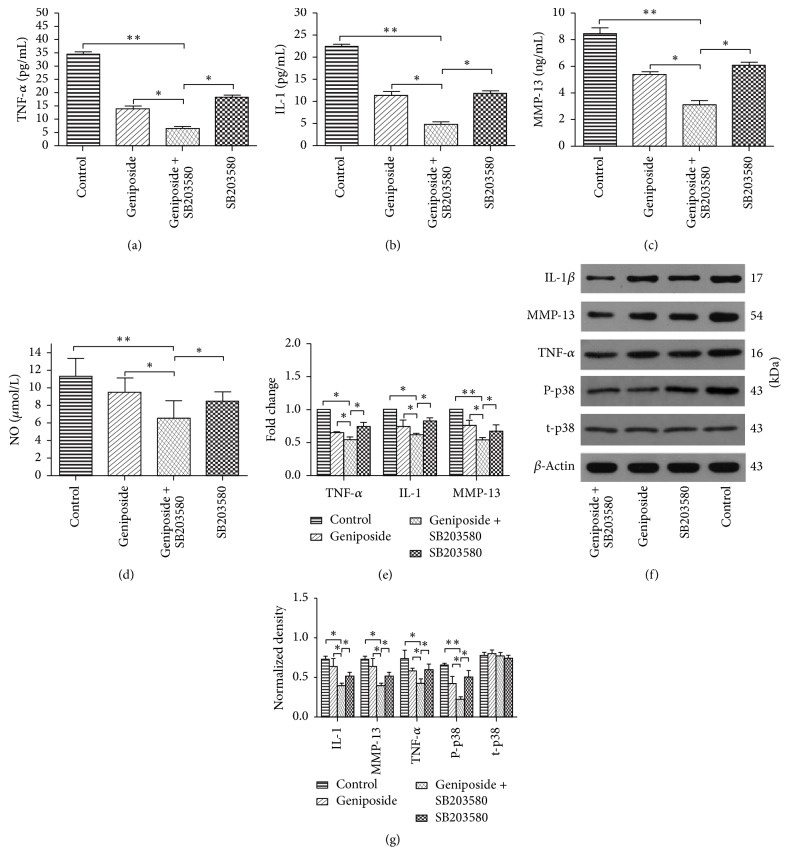
*Assessment of inflammatory mediators among different groups in vivo*. (a–d) The expression level of TNF-*α*, IL-1*β*, MMP-13, and NO in the synovial fluid of different groups treated with geniposide and SB203580 as well as the combination of these two drugs, respectively. (e) qRT-PCR analysis of IL-1*β*, TNF-*α*, and MMP-13 gene expression in the synovial fluid of the knee joint treated with geniposide, SB203580, and combination of these two drugs, respectively. The data of control were considered as 1. (f) Western blotting of IL-1*β*, TNF-*α*, MMP-13, p-p38 (phosphorylation of p38), and t-p38 (total p38) protein expression among four groups treated with blank (control), geniposide, SB203580, and combination of these two drugs, respectively. (g) Quantification of western blotting. Data is given as the mean ± SD; ^*∗*^*p* < 0.05 and ^*∗∗*^*p* < 0.01.

**Figure 5:**
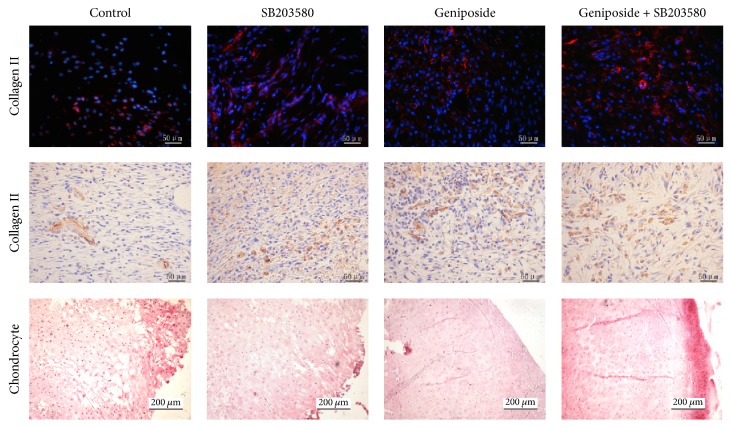
*Evaluation of cartilage*. The immunofluorescent staining and immunohistochemistry staining of collagen II in the cartilage of these four groups as well as the H&E staining of cartilage at day 14 after the intervention of the OA animals were shown.

By using ELISA, western blotting, and RT-PCR, we found that the expression and production of IL-1*β*, TNF-*α*, and MMP-13 in the OA knee joint were remarkably decreased in the group treated by geniposide coupled with SB203580 (Figures 4(a)–4(c), 4(e), 4(f), and 4(g)). Notably, the inhibitory effect of geniposide or SB203580 alone was also demonstrated (Figures 4(a)–4(c), 4(e), 4(f), and 4(g)). More importantly, the pathological damage of OA chondrocytes was observed among different groups (Figure 5). Specifically, the cartilage treated with geniposide combined with SB203580 exhibited a higher degree of well-organized chondrocytes, characterized by increased cells aggregation in a parallel arrangement as compared to the geniposide, SB203580, and control groups.

### 3.6. The Expression of NO in the Synovial Fluid of the Knee Joint

As shown in Figure 4(d), the levels of the cytokines NO were significantly lower in the GE + SB group (*p* < 0.001), indicating that GE may serve as a strong inhibitory agent to inhibit the NO production.

### 3.7. Effects of Geniposide on the Expression of Collagen II in the OA Chondrocytes

Compared with control group, OA chondrocytes of the group treated with geniposide and SB203580 showed increased expression of collagen II by using immunohistochemistry and immunofluorescence staining (Figure 5), implying the protective effect of geniposide on the OA chondrocytes.

## 4. Discussion

Although geniposide has been applied for the treatment of OA for years and the inhibitory effect of geniposide on the production of inflammatory cytokines has been demonstrated [17, 31, 32], few studies have investigated the specific mechanism of the application of geniposide on OA. This study clearly demonstrated that the inflammatory progress driven by the OA as well as the p38 MAPK pathway can be alleviated by geniposide. Thus, we concluded that the attenuation of p38 phosphorylation by geniposide appears to contribute to the inhibition of the inflammatory response in OA chondrocytes. This is a clinically beneficial finding, given that the application of most p38 inhibitors for OA has been thought to be limited due to their potential long-term toxicities [28].

Geniposide (GE) is the main bioactive component as an iridoid glycoside compound of* Gardenia* Ellis fruit. It has been widely used for treating rheumatism, knee pain, and traumatic bleeding by using its pharmacological property such as antihypertensive, antioxidative, and antiangiogenic effects [33, 34]. In addition, GE has also been shown to decrease the production of inflammatory factors including interleukin- (IL-) 4, IL-17, and TGF-beta-1 (TGF-*β*1) in peripheral blood lymphocytes and mesenteric lymph node (MLN) lymphocytes of arthritis rats [35]. However, few reports focused on the relation between geniposide and osteoarthritis, which is widely thought to be an inflammation related arthritis [5]. In this study, our results demonstrated the overexpression of inflammatory mediators including IL-1*β*, TNF-*α*, and MMP-13, which can be inhibited by geniposide in a dose-dependent manner. Our findings prove that geniposide may serve as an anti-inflammatory agent for OA.

Mitogen activated protein kinases (MAPKs), including c-Jun N-terminal kinases (JNK) and p38 MAPK signaling pathways, have been suggested to be extensively involved in regulating OA [36]. More importantly, activation of p38 MAPK has been reported to be highly associated with cartilage collagen degradation, chondrocyte apoptosis, and inflammation process in OA [37–39]. Thus, p38 MAPK has been considered to be an attractive target for drug-mediated modulation of inflammatory processes in OA [40]. This study clearly demonstrated that geniposide was able to downregulate the activation of p38 MAPK cascade (phosphorylation of the p38 signaling pathway)* in vitro* and* in vivo*, resulting in the alleviation of the expression and production of inflammatory mediators such as IL-1*β*, TNF-*α*, and MMP-13. Previously, Yamazaki et al. have proposed that genipin (the aglycone derived from geniposide) was able to induce neurite outgrowth by involving the NO-cGMP-PKG pathway with the help of nerve growth factor (NGF) [26]. However, the author did not determine whether and/or how PKG pathway could stimulate the MAPK cascade. We speculated that the discrepancy of the results may be attributed to the cell lines that the author used. PC12 cells, derived from rat pheochromocytoma, exhibit a variety of neuronal properties resembling those of sympathetic adrenal response, which are totally different from the chondrocytes.

SB203580, a classic inhibitor for p38 MAPK signaling pathway, has demonstrated its effective and reliable inhibition [41]. In this study, we utilized it as a reference to evaluate whether there is a synergistic effect when the geniposide and SB203580 are used together. Our findings showed that the inhibitory effect was remarkably amplified in both* in vitro* and* in vivo* test, indicating that the inhibition of geniposide on the production of inflammatory mediators was possibly by alleviating of activation of the p38 MAPK signaling pathway.

In this study, we found that geniposide was able to inhibit the expression and production of inflammatory mediators in OA chondrocytes and OA models. In addition, we demonstrated that this inhibitory effect was closely related to the alleviation of activation of the p38 MAPK signaling pathway by using a well-known p38 MAPK inhibitor SB203580 as a reference. Our findings provide evidence that geniposide may have therapeutic potential to serve as an alternative agent for the p38 MAPK inhibitor for the treatment of OA due to its inherent features of biological activities and low toxicity as a traditional Chinese medicine.

## 5. Conclusion

In summary, this study unambiguously shows that geniposide can inhibit the expression and production of inflammatory mediators in both OA chondrocytes and OA models. More importantly, our findings demonstrated that this inhibitory effect was closely related to the alleviation of activation of the p38 MAPK signaling pathway by using a well-known p38 MAPK inhibitor SB203580 as a reference. Our study concludes that geniposide may have therapeutic potential to serve as an alternative agent for the p38 MAPK inhibitor for the treatment of OA due to its inherent features of biological activities and low toxicity as a traditional Chinese medicine.

## Figures and Tables

**Table 1:** *The primer sequences for each primer used in the real-time RT-PCR*. The primer sequences and annealing temperature of IL-1*β*, TNF-*α*, MMP-13, and *α*-tubulin.

Genes	Primer sequences	Annealing temperature (°C)
*α*-Tubulin	Forward: GCTGTGGTTGAGCCCTACAAT	58
	Reverse: GTAGGTTGGGCGCTCAATGT	
IL-1*β*	Forward: TTGTTGAGCCAGGCCTCTCT	58
	Reverse: CCAAATGTGGCCGTGGTT	
MMP-13	Forward: CCCTCTGGTCTGTTGGCTCAC	58
	Reverse: CTGGCGTTTTGGGATGTTTAGA	
TNF-*α*	Forward: GGCTCAGAATCAGACCTCAG	58
	Reverse: GCTCCACATTGCAGAGAAGA	

## Abbreviations

MAPK: Mitogen activated protein kinase
OA: Osteoarthritis
TNF-*α*: Tumor necrosis factor
IL-1: Interleukin-1
NO: Nitric oxide
MMP: Matrix metalloproteinase
NSAIDs: Nonsteroidal anti-inflammatory drugs
GE: Geniposide
JNK: c-Jun N-terminal kinases
ERK: Extracellular signal-regulated kinase
COX-2: Cyclooxygenase-2
NGF: Nerve growth factor
ACL: Anterior cruciate ligament
OCT compound: Optimal Cutting Temperature compound
MTT: 3-(4,5-Dimethylthiazol-2-yl)-2,5-diphenyltetrazolium bromide
ELISA: Enzyme-linked immunosorbent assay
TGF: Transforming growth factor.
