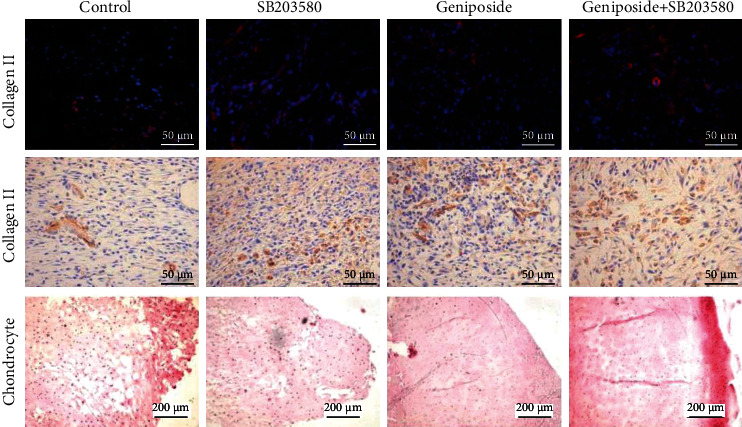# Corrigendum to “Anti-Inflammatory Effect of Geniposide on Osteoarthritis by Suppressing the Activation of p38MAPK Signaling Pathway”

**DOI:** 10.1155/2022/9814323

**Published:** 2022-01-10

**Authors:** Yuan Chen, Kangquan Shou, Chunlong Gong, Huarui Yang, Yi Yang, Tongzhu Bao

**Affiliations:** ^1^Department of Emergency and ICU, The First Clinical Medical College, China Three Gorges University, Yichang, Hubei 443002, China; ^2^Department of Orthopaedics, The First Clinical Medical College, China Three Gorges University, Yichang, Hubei 443002, China

In the article titled “Anti-Inflammatory Effect of Geniposide on Osteoarthritis by Suppressing the Activation of p38MAPK Signaling Pathway” [1], an image duplication was identified in Figure 5. The authors explained that this was due to an error introduced during the preparation of the manuscript and provided the correct panels as below.

## Figures and Tables

**Figure 1:**